# Intrusive memories to traumatic footage: the neural basis of their encoding and involuntary recall

**DOI:** 10.1017/S0033291715002007

**Published:** 2016-02

**Authors:** I. A. Clark, E. A. Holmes, M. W. Woolrich, C. E. Mackay

**Affiliations:** 1 Department of Psychiatry, University of Oxford, Warneford Hospital, Oxford OX3 7NG, UK; 2 Medical Research Council Cognition and Brain Sciences Unit, 15 Chaucer Road, Cambridge CB2 7EF, UK; 3 Division of Psychology, Department of Clinical Neuroscience, Karolinska Institutet, Stockholm, Sweden; 4 Department of Psychiatry, Oxford Centre for Human Brain Activity (OHBA), Warneford Hospital, Oxford OX3 7NG, UK

**Keywords:** Dorsolateral prefrontal cortex, flashbacks, functional magnetic resonance imaging, intrusive memories, left inferior frontal gyrus

## Abstract

**Background:**

A hallmark symptom after psychological trauma is the presence of intrusive memories. It is unclear why only some moments of trauma become intrusive, and how these memories involuntarily return to mind. Understanding the neural mechanisms involved in the encoding and involuntary recall of intrusive memories may elucidate these questions.

**Method:**

Participants (*n* = 35) underwent functional magnetic resonance imaging (fMRI) while being exposed to traumatic film footage. After film viewing, participants indicated within the scanner, while undergoing fMRI, if they experienced an intrusive memory of the film. Further intrusive memories in daily life were recorded for 7 days. After 7 days, participants completed a recognition memory test. Intrusive memory encoding was captured by comparing activity at the time of viewing ‘Intrusive scenes’ (scenes recalled involuntarily), ‘Control scenes’ (scenes never recalled involuntarily) and ‘Potential scenes’ (scenes recalled involuntarily by others but not that individual). Signal change associated with intrusive memory involuntary recall was modelled using finite impulse response basis functions.

**Results:**

We found a widespread pattern of increased activation for Intrusive *v*. both Potential and Control scenes at encoding. The left inferior frontal gyrus and middle temporal gyrus showed increased activity in Intrusive scenes compared with Potential scenes, but not in Intrusive scenes compared with Control scenes. This pattern of activation persisted when taking recognition memory performance into account. Intrusive memory involuntary recall was characterized by activity in frontal regions, notably the left inferior frontal gyrus.

**Conclusions:**

The left inferior frontal gyrus may be implicated in both the encoding and involuntary recall of intrusive memories.

## Introduction

The majority of people will experience or witness a traumatic event during their lifetime and a significant minority will develop post-traumatic stress disorder (PTSD) (Breslau *et al.* 1998; American Psychiatric Association, 2013). A hallmark symptom of PTSD is the occurrence of intrusive memories – involuntary images of the trauma intruding into consciousness (Brewin, 2013). We lack understanding of why only some moments within a trauma are (re)experienced as intrusive memories and how these moments involuntarily return to mind. Processing at the time of trauma (peritraumatic processing) – i.e. during memory encoding – has been implicated in both later PTSD and intrusive memory development (Ehlers & Clark, 2000; Ozer *et al.* 2003; American Psychiatric Association, 2013; Brewin, 2014). Investigating the neural mechanisms during encoding may add to our understanding of intrusive memories. The current study investigated a hypothesized neural ‘signature’ during the encoding of an experimental analogue of trauma (Bourne *et al.* 2013), and the involvement of this signature in later intrusive memory involuntary recall.

Due to the nature of PTSD, the wealth of neuroimaging work has been conducted in PTSD patients with established symptoms (Lanius *et al.* 2006; Hughes & Shin, 2011). Such research has often used symptom provocation paradigms, which involve exposing PTSD patients to reminders of their trauma while undergoing neuroimaging (Rauch *et al.* 1996; Shin *et al.* 1997, 2004, 2006; Lanius *et al.* 2006; Hughes & Shin, 2011). Neurocircuitry models from this work suggest that PTSD is characterized by increased amygdala (and other limbic) activation and reduced ventromedial prefrontal cortex activation (Rauch *et al.* 1998, 2006). A recent model by Admon *et al*. (2013) suggested that abnormalities in the amygdala and dorsal anterior cingulate cortex are predisposing, while abnormal interactions between the hippocampus and ventromedial prefrontal cortex arise after developing PTSD (Admon *et al.* 2013). However, patient studies can tell us little about how intrusive memories are formed since they cannot examine the original encoding of the trauma.

Studying the neural correlates of real-life trauma is unfeasible, but intrusive memories can be experimentally induced using an experimental analogue – the trauma film paradigm (Lazarus, 1964; Holmes & Bourne, 2008). Participants view footage of real-life scenes of death and serious injury, in line with the fifth edition of the Diagnostic and Statistical Manual of Mental Disorders (DSM-5; American Psychiatric Association, 2013) definition for psychological trauma. Combining the trauma film with neuroimaging allows a prospective design to study intrusive memory encoding and involuntary recall.

We recently conducted, to our knowledge, the only study to date investigating the neural basis of intrusive memory encoding (Bourne *et al.* 2013). Results suggested a widespread neural signature at the time of viewing scenes that later became intrusive memories, including increases in activation in the amygdala, striatum, rostral anterior cingulate cortex, thalamus and ventral occipital cortex.

In particular, two regions (and only these) seemed to distinguish between scenes that became intrusive memories for an individual and scenes that had the ‘potential’ to become intrusive memories (i.e. scenes of emotional content recalled involuntarily by some participants, but not that individual); the left inferior frontal gyrus (IFG) and middle temporal gyrus (MTG) (Bourne *et al.* 2013). These results at encoding partially mirror the ‘subsequent memory effect’ found in non-traumatic memory (Paller & Wagner, 2002; Kensinger & Corkin, 2004). The subsequent memory effect suggests predictive differences at encoding for items that are later deliberately recalled relative to items that are not recalled in left prefrontal regions and bilateral middle temporal regions – areas that include the left IFG and MTG. We therefore sought to ask whether these two regions would also predict moments of the film that would be recalled involuntarily. Another possible explanation for our previous results is that intrusive memory scenes were simply better recognized than potential scenes. These encoding results require replication, and additionally for recognition memory to be taken into account.

Our second question concerns the neural basis of intrusive memory involuntary recall. To our knowledge, no study has captured the neural activation at the moment of intrusive memory involuntary recall – that is, the moment when a participant experiences an intrusive memory while undergoing functional magnetic resonance imaging (fMRI). Symptom provocation studies indicate increased activity in limbic and paralimbic areas, suggesting that these regions may be involved in intrusive memory involuntary recall (Rauch *et al.* 1996; Liberzon *et al.* 1997; Shin *et al.* 1999; Osuch *et al.* 2001). However, while patients may (or may not) experience intrusive memories during scanning, the neural mechanisms of their involuntary recall remain unknown as an intrusive memory could have occurred at any point during symptom provocation. Further, other symptoms with different underpinning may be implicated during symptom provocation (Bryant *et al.* 2013; Pietrzak *et al.* 2013). In a separate vein, one study using healthy participants has shown that the involuntary recall of picture stimuli, compared with their voluntary recall, has been associated with the middle and superior frontal gyri (Hall *et al.* 2008). Whether these results can be extrapolated to intrusive memories of traumatic stimuli is unknown.

We note that part of the data acquired and presented here (the fMRI data concerning encoding) is also used elsewhere (Clark *et al.* 2014) in combination with our previous work (Bourne *et al.* 2013). Using different analysis techniques we attempted to investigate a second separate question – one of prediction instead of association. That is, could we ‘learn’ the brain activity associated with later intrusive memories in order to predict, from new unseen brain activity, ‘future’ intrusive memories? On the other hand, here, we report the differences in brain activity during scenes that were later recalled involuntarily by that participant (intrusive scenes), compared with scenes recalled involuntarily by previous participants, but not that individual (potential scenes). The work presented in the present paper therefore attempts to identify regions that may differentiate between intrusive and potential scenes. Our parallel work (Clark *et al.* 2014) attempts to quantify the extent that solely the peritraumatic brain activity can predict intrusive memories. Thus, although we recognize that we use the same component of the dataset in two different papers, we use it to address two distinctly different questions.

The current experiment investigated the encoding and involuntary recall of intrusive memories of experimental trauma. We first sought to replicate our previous findings of widespread increases in neural activation at the time of viewing scenes that caused intrusive memories relative to scenes that did not. Specifically, we predicted that activation in the left IFG and MTG would distinguish intrusive memory scenes from ‘Potential’ scenes (scenes of emotional content recalled involuntarily by previous participants, but not that individual). Further, to take into account possible signal changes due to better recognition memory for intrusive compared with potential scenes, we reconfigured the fMRI time series into film ‘stills’ to repeat our encoding analysis using only correctly recognized film picture stills. Finally, we sought to investigate the neural mechanisms of intrusive memory involuntary recall, modelling brain activity while participants experienced an intrusive memory during fMRI. To adaptively capture the moment of involuntary recall we modelled the fMRI time series data using finite impulse response basis functions.

## Method

### Participants

A total of 41 participants were recruited from the local community. Data could not be analysed for six participants (online Supplementary material). This left 35 participants (mean age = 22.43 years, s.d. = 7.52; 29 female, six male) with no reported current or previous psychiatric history. The study was approved by the University of Oxford Central University Research Ethics Committee. All participants provided written informed consent and were reimbursed £25 (US $40).

### Behavioural measures

#### Trauma film viewing

**Fig. 1.**
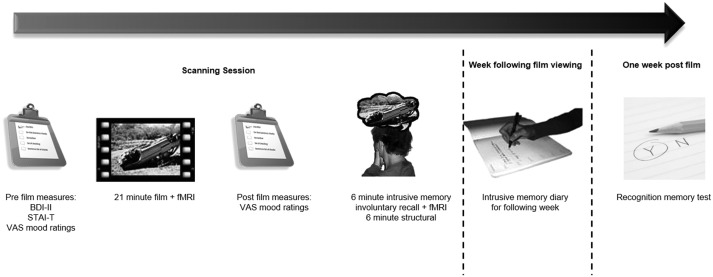
Experimental procedure. Participants completed baseline questionnaires and measures of their current mood. They then viewed film footage with traumatic content, including scenes of death and serious injury, while undergoing functional magnetic resonance imaging (fMRI). On film completion participants were removed from the scanner and mood measurements were administered. Participants were then trained to identify intrusive memories. They were then returned to the scanner indicating with a button press if they experienced an intrusive memory of the film while undergoing fMRI. For the following week participants kept a diary of any further intrusive memories, returning at 1 week to perform a recognition memory test of the film contents. BDI-II, Beck Depression Inventory-II; STAI-T, State–Trait Anxiety Inventory, trait scale; VAS, visual analogue scale.

The experimental procedure is shown in Fig. 1. After completing baseline and mood measures (online Supplementary material) participants viewed traumatic film footage, including scenes of actual and threatened death and serious injury, while undergoing fMRI. The film comprised 15 short clips which included 20 Possible intrusive scenes and 16 Control scenes. Scene type was determined using data from approximately 200 participants who had taken part in previous behavioural experiments. ‘Possible’ scenes were scenes that had induced intrusive memories in previous participants (e.g. emergency personnel at an accident with an injured victim), ‘Control’ scenes were those that had never induced intrusive memories (e.g. emergency personnel around the accident but no visible death or injury). Possible scenes were later classified as either ‘Intrusive’ scenes (recalled involuntarily by that participant) or ‘Potential’ scenes (not recalled involuntarily by that participant, but recalled involuntarily by previous participants) depending on the diary data (see Intrusive memory diary below). All scenes had unique topic content to facilitate intrusive memory identification. Scene length was matched as closely as possible between Possible (length, 5–37 s; mean 22.5 s) and Control scenes (length, 5–36 s; mean 16.4 s) (*t*_34_ = 1.94, n.s.); see the online Supplementary material (online Supplementary Tables S1 and S2) for the exact duration of each scene. Scenes were distributed evenly throughout the whole film. These constraints were included to take into account the relative slowness of the haemodynamic response (Buxton *et al.* 2004).

#### Intrusive memory involuntary recall during fMRI

On film completion, participants were briefly removed from the scanner to complete mood ratings. As per previous trauma film paradigm experiments (Holmes *et al.* 2004), participants were instructed as how to identify intrusive memories – defined as: (1) moments of the film spontaneously popping into mind unexpectedly (rather than the participant purposefully recalling the film); and (2) mental images, e.g. taking the form of pictures or sounds.

Participants then returned into the scanner. Participants were asked to lie in the scanner for 6 min and respond with a button press if they experienced an intrusive memory of the film (i.e. if the film spontaneously popped into their mind). To minimize experimental demands, it was made clear to the participants that they may – or may not – experience any intrusive memories during the scan. This allowed us to capture the moment of intrusive memory involuntary recall while participants were in the scanner undergoing fMRI.

#### Intrusive memory diary

Participants kept a daily intrusive memory diary for the following week (Holmes *et al.* 2004). Participants were asked to write the content of any intrusive memory (e.g. the car hitting the boy) and their emotional rating of the intrusive memory, from 1 ‘very negative’ to 5 ‘very positive’. All content descriptions were checked to confirm they matched a specific film scene. Intrusive memories experienced during the intrusive memory involuntary recall scan were included in the diary. From the diary Intrusive and Potential scenes we derived from the Possible scenes retrospectively for each participant. That is, ‘Intrusive’ scenes were those matched with the involuntary memories reported in the diary, and ‘Potential’ scenes were the remaining Possible scenes that did not return as intrusive memories for that participant.

#### Film still recognition memory test

At 1 week post-film viewing participants performed a yes/no recognition memory test containing 201 picture stills; 103 from the film (51 from Control scenes, 52 from Potential and Intrusive scenes) and 98 foils – see the online Supplementary material.

### fMRI data acquisition

All fMRI imaging data (trauma film viewing and intrusive memory involuntary recall) were acquired on a 3-T Siemens TIM Trio System with a 12-channel head coil (voxel resolution = 3 × 3 × 3 mm^3^; repetition time = 3 s; echo time = 30 ms). T1-weighted structural images were acquired for subject registration using a magnetization prepared rapid gradient echo (MPRAGE) sequence (voxel resolution = 1 × 1 × 1 mm^3^; repetition time = 2040 ms; echo time = 4.7 ms).

### fMRI data analysis

Analyses were performed using FEAT (fMRI Expert Analysis Tool) version 6.0 (http://www.fmrib.ox.ac.uk/fsl). Data were pre-processed using FEAT's default options: motion correction applied using MCFLIRT and fieldmaps with an echo planar imaging (EPI) spacing of 0.49 ms and echo time of 22 ms; Gaussian spatial smoothing applied with a full width half maximum of 5 mm; brain matter separated from non-brain using a mesh deformation approach; high-pass temporal filtering applied with a cut-off of 100 s.

#### Intrusive memory encoding

Analysis was performed at a whole-brain level. The three event types (Intrusive, Potential, Control) were specified for each participant in the general linear model with a fourth variable of no interest to model text slides (which provided information concerning each film clip). The model was applied voxel-wise to the pre-processed imaging data. First-level within-subject analysis was performed using FILM (FMRIB's Improved Linear Model). Voxel-wise group analysis was performed in Montreal Neurological Institute (MNI) 152 standard space using FLAME (FMRIB's Local Analysis of Mixed Effects) stage 1 (Beckmann *et al.* 2003; Woolrich *et al.* 2004; Woolrich, 2008). *z-*Statistic images were thresholded at *z* > 2.3 and a family-wise error corrected cluster significance threshold of *p* < 0.05 (Forman *et al.* 1995).

Following whole-brain analysis, percentage blood oxygen level-dependent (BOLD) signal change was extracted from the left IFG and MTG using predefined regions of interest (ROIs). The left IFG and MTG were defined as regions that were significantly activated in the Bourne *et al.* (2013) results on a whole-brain basis in the Intrusive (referred to as Flashback) *v*. Potential contrast, but not for the Intrusive *v*. Control contrast.

To control for any effect of Intrusive scenes being better recognized than Potential scenes we performed an additional analysis (as above) using only Intrusive recognized picture stills and Potential recognized picture stills (identified by the recognition memory test) with each still modelled for 0.5 s.

#### Intrusive memory involuntary recall

We recruited nine additional participants to act as a control group (online Supplementary material). Participants underwent a 6 min scan randomly pressing a button approximately 5–10 times.

Analysis was performed at the whole-brain level. For both groups (intrusive memory involuntary recall and control button press) 3-s wide (the repetition time) finite impulse response (FIR) basis functions modelled consecutive ‘time bins’ surrounding the button press (Diederen *et al.* 2010). To take into account the approximate 6 s delay in haemodynamic response, the time bins were placed from −3 to +12 s in relation to the button press – resulting in five time bins for each button press. The five time bins were entered into a single general linear model and applied to the pre-processed data in FILM for each participant. The FIR basis function was modelled as a single basis function with a 0 s phase shift and 3 s time window. Exploratory group-wise analysis was performed at the whole-brain level in MNI standard space using FLAME with *z* statistic images thresholded at *z* > 1.7 and a family-wise error-corrected cluster significance threshold of *p* < 0.05.

Following whole-brain analysis, percentage BOLD signal change was extracted from ROIs showing significant activation in the intrusive memory involuntary recall *v*. control button press contrast and the reverse contrast in any of the time bins. Additional signal change was extracted from the precentral gyrus to compare motor activity. The precentral gyrus was created from the Oxford–Harvard cortical and subcortical probabilistic anatomical atlas thresholded at a minimum probability of 20%.

### Ethical standards

All procedures contributing to this work comply with the ethical standards of the relevant national and institutional committees on human experimentation and with the Helsinki Declaration of 1975, as revised in 2008.

## Results

### Behavioural results

Baseline measures and mood change are reported in the online Supplementary material.

In terms of the main outcome of interest, participants reported a total of 303 intrusive memories that could be matched in content to the film from the 1-week diary and in the scanner soon after film viewing (mean per participant = 8.66, s.d. = 7.15). A further 13 intrusive memories could not be matched to the film (95.9% did match) and were not included in analyses. The mean emotion rating of the intrusive memories was 2.15 (s.d. = 0.45), suggesting that participants found their intrusive memories negative.

The number of different scene types per person was the variable of interest for the fMRI analysis. The mean number of Intrusive scenes per participant was 3.09 (s.d. = 1.46), leaving a mean number of Potential scenes (from the 20 possible scenes) of 16.91 (s.d. = 1.46). The number of Control scenes was pre-determined at 16 per participant.

On the recognition memory test at 1 week, in our set, Intrusive picture stills were better recognized than Potential picture stills (83.04%, s.d. = 13.89; 64.07%, s.d. = 14.97, respectively; *t*_34_ = 6.76, *p* < 0.001). For further information, see the online Supplementary material.

In the scanner soon after film viewing, 25 participants reported intrusive memories of the film, totalling 148 intrusive memories (mean frequency = 5.92, s.d. = 4.08; mean number different intrusive memory scenes = 2.36, s.d. = 1.37). The nine control participants had a mean number of button presses of 7.89 (s.d. = 2.15).

### fMRI results

#### Intrusive memory encoding

**Fig. 2.**
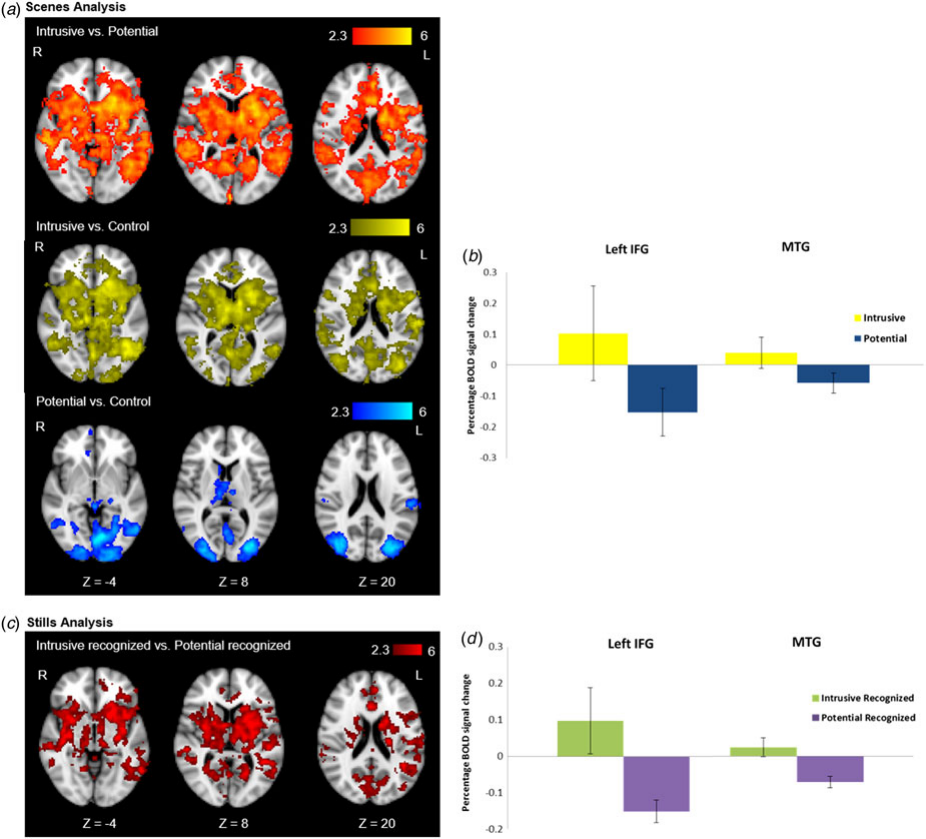
Neural basis of intrusive memory encoding. (*a*) Whole-brain analysis of the encoding of Intrusive *v*. Potential *v*. Control scenes, increased blood oxygen level-dependent (BOLD) responses in colour for each contrast. (*b*) Region-of-interest (ROI) analysis for the left inferior frontal gyrus (IFG) and middle temporal gyrus (MTG) showing the BOLD percentage signal change for Intrusive and Potential scenes relative to Control scenes. (*c*) Whole-brain analysis of the encoding of Intrusive recognized *v*. Potential recognized, increased BOLD response shown in colour. (*d*) ROI analysis for the left IFG and MTG showing the BOLD percentage signal change for Intrusive recognized and Potential recognized picture stills. Values are means, with standard deviations represented by vertical bars. R, Right; L, left.

Whole-brain analysis comparing Intrusive with Potential (Fig. 2*a*, top row) and Control scenes (Fig. 2*a*, middle row) revealed widespread increases in activation, including the putamen, rostral anterior cingulate cortex, insula, thalamus and ventral occipital cortex. Signal change extracted from predefined ROIs showed, as predicted, differences in activation in the MTG and left IFG between Intrusive and Potential scenes but not between Intrusive and Control scenes (Fig. 2*b*). Comparison of Potential scenes with Control scenes at the whole-brain level (Fig. 2*a* bottom row) revealed increased activation in the thalamus and ventral occipital cortex. Table 1 shows peak voxel coordinates.

**Table 1.** Peak voxel coordinates identified in the whole-brain intrusive memory encoding analysis^a^

Analysis	Location	Cluster size	*x*	*y*	*z*	*z*-Statistic
Intrusive > potential	Left putamen	80 991	−14	8	2	6.08
	Left precuneus		−22	−54	6	5.94
	Left insular		−36	12	−6	5.83
	Left middle temporal gyrus		−56	−56	10	5.82
	Left caudate		−12	14	0	5.81
Intrusive > control	Left inferior temporal gyrus	85 347	−44	−58	−6	6.37
	Left supramarginal		−60	−26	30	6.27
	Left caudate		−14	10	2	6.25
	Right thalamus		10	−20	10	6.18
Potential > control	Left occipital fusiform gyrus	30 148	−20	−70	−14	6.51
	Right supramarginal		60	−22	36	6.48
	Left lingual gyrus		−8	−72	−6	6.45
	Left temporal occipital fusiform cortex		−26	−62	−12	6.44
	Right superior parietal		22	−50	74	6.2
	Right thalamus	939	8	−16	12	5.18
	Left thalamus		−8	−14	12	4.14
	Right caudate		12	0	12	4.09
	Right superior frontal	769	24	−6	62	5.28
	Right middle frontal		26	−2	54	4.71
	Left superior frontal	625	−26	−4	62	4.33
	Left precentral		−22	−10	66	3.48
	Right frontal pole	572	14	56	−14	4.43
	Right frontal orbital		22	34	−12	3.81
	Brain stem	528	0	−34	−4	4.51
	Left thalamus		−22	−26	−4	3.44
	Posterior cingulate		−2	−38	4	3.42
	Hippocampus		−6	−38	6	3.25
Intrusive recognized > potential recognized	Left putamen	40 032	−20	8	0	4.95
	Left frontal orbital		−28	16	−18	4.84
	Right putamen		30	−16	2	4.82

a Brain regions identified using the Oxford–Harvard cortical and subcortical anatomical atlas.

Behavioural results on the recognition memory test highlighted that Intrusive picture stills were better recognized than Potential picture stills. Further comparison of the neuroimaging data was therefore made using only those Intrusive and Potential picture stills correctly recognized as from the film to control for any neural effects that could be explained by this better recognition memory. Results show a similar pattern of activation as to the original Intrusive *v*. Potential analysis at both whole-brain (Fig. 2*c*) and ROI level (Fig. 2*d*). Table 1 shows peak voxel coordinates. See also online Supplementary material and Fig. S1.

#### Intrusive memory involuntary recall

**Fig. 3.**
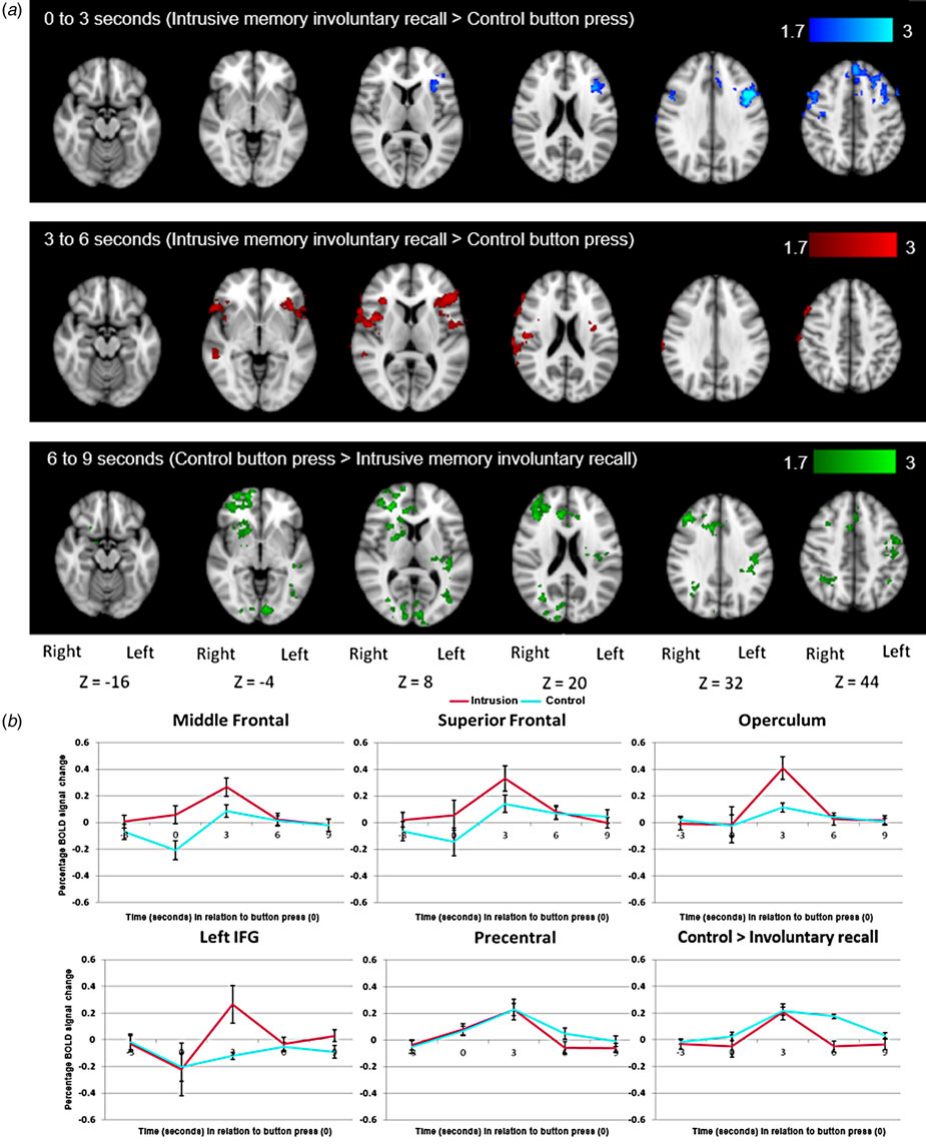
Intrusive memory involuntary recall. (*a*) Whole-brain analysis showing the increased blood oxygen level-dependent (BOLD) response for intrusive memory involuntary recall *v*. control button press group at the two time bins (0–3 s and 3–6 s in relation to the button press) showing significant differences in activation, and the one time bin (6–9 s) showing increased BOLD response for the control button press group *v*. intrusive memory involuntary recall. (*b*) Region-of-interest profile plots of the signal change observed across each time bin from −3 to +12 s in relation to the button press. Intrusive memory involuntary recall signal change activation is shown in pink, control button press signal change activation in light blue. Values are means, with standard deviations represented by vertical bars. IFG, Inferior frontal gyrus.

Whole-brain analyses using the five time bins compared activation during intrusive memory involuntary recall (i.e. involuntary recall in the scanner soon after film viewing) and the control button press (Fig. 3*a*; peak voxel coordinates in Table 2). Increased activation for the intrusive memory involuntary recall group compared with the control button press group was seen in middle and superior frontal regions between 0 and 3 s and in the left IFG and bilateral operculum between 3 and 6 s. Increased activation for the reverse contrast was seen between 6 and 9 s. No suprathreshold activation was found for either contrast between −3 and 0, and 9–12 s.

**Table 2.** Peak voxel coordinates identified in the whole-brain intrusive memory involuntary recall analysis^a^

Analysis	Location	Cluster size	*x*	*y*	*Z*	*z*-Statistic
Intrusion > control; 0–3 s	Left middle frontal gyrus	4312	−48	14	32	3.39
	Right postcentral		54	−10	54	3.12
	Superior frontal		0	10	60	3.09
	Right precentral		44	−12	68	3.06
Intrusion > control; 3–6 s	Right inferior frontal gyrus	2997	60	14	2	3.4
	Right postcentral		58	−12	54	3.21
	Right middle frontal		54	18	42	3.05
	Right central opercular		36	6	16	2.96
	Left frontal operculum	1205	−32	22	10	3.12
	Left inferior frontal		−48	30	8	2.78
	Left central opercular		−46	−2	14	2.74
	Left frontal orbital		−40	22	−4	2.68
	Left inferior frontal		−52	36	6	2.68
Control > intrusion; 6–9 s	Right middle frontal gyrus	8180	42	34	28	3.24
	Right frontal pole		30	42	10	2.92
	Right postcentral		26	−30	64	2.87
	Left lingual gyrus	2324	−6	−86	−6	2.71
	Right supracalcarine cortex		6	−78	16	2.66
	Right superior parietal		20	−56	72	2.6
	Right lateral occipital		32	−66	52	2.55
	Right intracalcarine cortex		14	−82	12	2.53

a Brain regions identified using the Oxford–Harvard cortical and subcortical anatomical atlas.

Signal change was extracted from ROIs showing significant activation at the whole-brain level and the precentral gyrus. BOLD signal activation profile plots are shown in Fig. 3*b*. The BOLD signal response in the precentral gyrus followed the same pattern in both groups; peaking during the time bin modelling 3–6 s after the button press. This peak (expected due to the delay in BOLD signal response) suggests successful modelling of changes in activation by the finite impulse response basis functions.

## Discussion

The current study investigated the neural basis of the encoding and involuntary recall of intrusive memories to film footage of traumatic events. We found a widespread pattern of increased activation at the encoding of Intrusive scenes (emotional scenes that were involuntarily recalled) compared with both Potential scenes (emotional scenes that were not involuntarily recalled by that participant but were previously by other participants) and Control scenes (scenes that were never involuntarily recalled). As predicted, the left IFG and MTG showed increased activity between Intrusive *v*. Potential scenes, but not Intrusive *v*. Control scenes. These areas may potentially distinguish whether a scene which is traumatic in content will intrude or not.

The encoding findings provide a strong replication of our previous work (Bourne *et al.* 2013) in a new sample. In addition to the previous work and importantly, we were able to show that while Intrusive picture stills were better recognized than Potential picture stills, analysis using only recognized picture stills revealed the same pattern of brain activation.

The fMRI involuntary recall findings captured the neural activation at the moment of intrusive memory involuntary recall (that is, experiencing an intrusive memory in the scanner) indicating involvement of the middle and superior frontal cortices, operculum and left IFG. The left IFG was the only region to be involved in both intrusive memory encoding and involuntary recall.

### Intrusive memory encoding

Results provide support for a neural signature at the time of viewing those scenes from footage of traumatic events that later return as an intrusive memory. By comparing activation during Intrusive and Potential picture stills that were recognized at 1 week, we are able to suggest that the differential activation between Intrusive and Potential scenes was not merely due to participants having better recognition memory for Intrusive scenes. That is, we know that all the picture stills used in the analysis were recognized by participants. Neural activation found from comparing intrusive and potential events can therefore not simply be explained as an indication of better recognition memory performance for intrusive events. Indeed, the similarity in brain regions identified by both contrasts supports the notion that neural signature is associated with the later involuntary recall of that event *per se*.

Multiple regions were associated with the encoding of Intrusive scenes, and included, but were not limited to, regions associated with threat detection, in particular surprise to threat (e.g. rostral anterior cingulate cortex) (Bishop *et al.* 2004; Browning & Harmer, 2012), emotional processing, pain and empathy of pain (e.g. insula, anterior cingulate, thalamus) (Leknes & Tracey, 2008) and visual processing and mental imagery (e.g. ventral occipital cortex) (Kosslyn *et al.* 2001). These are regions consistent with intrusive memories being emotional, vivid images of traumatic events and regions that are partially in line with models of PTSD (Rauch *et al.* 2006; Admon *et al.* 2013).

What might be the theory underlying our neural activation results in the creation of an intrusive memory in comparison with a traumatic moment that does not later return involuntarily? While our experimental design restricts conclusions, we speculate on the following as a starting point for future theoretical development. Neurocircuitry models of PTSD draw on animal fear-conditioning models and implicate emotional regions such as the limbic system (e.g. Rauch *et al.* 2006). Wegerer *et al.* (2013) have argued that fear conditioning also underlies intrusive memories, albeit in behavioural studies. While our results do highlight emotion regions, in line with fear-conditioning models, a number of additional regions were also identified in our study (e.g. MTG and IFG). This indicates that additional processing beyond that of fear conditioning may be involved (see Beckers *et al.* 2013).

Several other literatures also provide theoretical insights. Models of intrusive memories in PTSD treatment stemming from clinical and cognitive psychology implicate emotional regions, and additionally point to heightened activity in sensory/imagery-related regions (suggested to be mediated by the precuneus) alongside decreased activity in memory regions (Brewin *et al.* 2010; Brewin, 2014). This is proposed to lead to ineffective coupling of emotional and contextual information and thus the later occurrence of intrusive memories. Our results are partially consistent with this model (e.g. occipital areas), supporting the emphasis on mental imagery. Notably, imagery is not mentioned in the above neurocircuitry models of PTSD (Rauch *et al.* 2006). However, we argue that the emphasis on imagery should not be restricted to PTSD memory recall, but rather is part of a continuum with non-clinical autobiographical recall. Episodic memory involves imagery (Tulving, 2002). Vivid image-based autobiographical memories have been associated with activity in occipital regions and the precuneus (Cabeza & St. Jacques, 2007), and the underlying neural processes associated with mental imagery substantially overlap with those for autobiographical memory (Hassabis & Maguire, 2007; Schacter & Addis, 2007). This link between autobiographical memory and intrusive memories is also underscored by autobiographical memory theorists who span clinical and non-clinical literatures (Conway, 2001; Berntsen & Hall, 2004; Rubin *et al.* 2008).

Where our results notably differ from previous models of intrusive memories and PTSD is the activity pattern found in the left IFG and MTG. The left IFG and MTG showed increased activity between Intrusive and Potential scenes, but not Intrusive and Control scenes. We suggest that these brain regions may be involved in distinguishing why particular traumatic scenes become an intrusive memory while other traumatic scenes in the same sequence do not. As noted in the introduction, both regions have previously been associated with subsequent memory for deliberate recall (Paller & Wagner, 2002; Kensinger & Corkin, 2004). We suggest that enhanced encoding occurs at these ‘hotspot’ moments which later become intrusive memories, with heightened involvement of these memory-related areas in combination with increases in sensory and emotional processing. In contrast, PTSD models proposed elsewhere suggest ‘disrupted’ encoding and memory fragmentation (e.g. Brewin, 2014).

### Intrusive memory involuntary recall during fMRI

Our final aim of the study was to model brain activity when participants experienced an intrusive memory in the scanner while undergoing fMRI. Using finite impulse response basis functions to model the BOLD signal change we identified neural activity at the moment of intrusive memory involuntary recall. Initial activity was observed in the middle and superior frontal cortices, followed by activation in the operculum and left IFG. These findings of middle and superior frontal cortex activity are convergent with previous results of involuntary recall for picture stimuli (Hall *et al.* 2008), extending this previous finding to the involuntary recall of more naturalistic complex film stimuli. In PTSD patients, decreases in activity following treatment in the middle frontal cortex during trauma imagery have also been identified (Lindauer *et al.* 2008). Additionally, the frontal operculum has been associated with the attentional control of cognitive processes and task selection (Higo *et al.* 2011) and the left IFG with the selection of competing memory representations (Nelson *et al.* 2009; Levens & Phelps, 2010).

### The left IFG and intrusive memories

The left IFG was the only region identified here involved in both intrusive memory encoding and involuntary recall. Interestingly, studies of PTSD patients have indicated neural networks involving the left IFG (James *et al.* 2013) and the left IFG has shown increases in activity when PTSD patients process traumatic compared with neutral material (Landré *et al.* 2012). Current neurocircuitry models do not implicate the left IFG in PTSD (Rauch *et al.* 2006; Admon *et al.* 2013), though we note that this study involves encoding, which by definition has not been examined in PTSD patients.

What might be the role of the left IFG in intrusive memory encoding and recall, at least for experimental trauma? As previously mentioned, the left IFG has been associated with predicting later subsequent memory recall (Kensinger & Corkin, 2004). It has also been associated with the selection of information (Moss *et al.* 2005), competing memory representations (Nelson *et al.* 2009; Levens & Phelps, 2010) and evaluations of emotional information (Lee & Siegle, 2012). A meta-analysis of cognitive control suggests that the left IFG may be involved in the ‘flexibility’ to switch from one task to another (Niendam *et al.* 2012). Further, greater putamen–left IFG functional connectivity activity has been associated with unwanted thoughts in healthy participants (Kühn *et al.* 2014). We do note, however, that these associations, while interesting, are made with reverse inference and thus should be done so with caution (Poldrack, 2006).

From our current results, we tentatively hypothesize that left IFG activation while viewing traumatic material (during encoding) may ‘flag’ the event that will subsequently return as an intrusive memory, comprising an analogue trauma ‘hotspot’ (Grey & Holmes, 2008). During intrusive memory involuntary recall, left IFG activation may represent the orientation of attention towards the ‘flagged’ memory, contributing to the overriding of other psychological functioning and capture of attention.

Overall, our view is that trauma intrusions are not simply bits of ‘fragmented or incoherent’ memory, rather that intrusions comprise highly selective hotspots meaningful to that individual (Ehlers *et al.* 2002; Grey & Holmes, 2008; Krans *et al.* 2009). Further we see intrusive memory in PTSD on a continuum with other emotional intrusive memories, and on a continuum between clinical and non-clinical populations (see also Kvavilashvili, 2014).

### Limitations

The number of events modelled in the current experiment is low compared with more traditional fMRI designs, though similar to the number of different intrusive memories seen in PTSD patients – a mean of 3.74 (Grey & Holmes, 2008). This may be inevitable in paradigms attempting to capture ‘rare’ clinically relevant symptoms. Conventional ideas may suggest that three events provide insufficient power for a reliable contrast. On the other hand, the statistics used do account for the low number of events, and a small number of events is more likely to cause a type II error (false negative) than a type I error (false positive), because low event frequency increases noise, making it difficult to find meaningful results (see also empirical demonstration in Bourne *et al.* 2013; online Supplementary material).

Due to the nature of the traumatic content, scene length varied between 5 and 37 s. This is greater variance than is typically seen in fMRI study designs. Given the slowness of the haemodynamic response (between 5 and 7 s), this adds further noise to the data. Further, the total time modelled as specific scenes of interest is relatively low (indeed, the total scan time of the whole film is under 25 min). Conventional wisdom suggests scanning for as long as possible and collecting the most data over events of interest as possible, resulting in longer scan times and greater amounts of data (see, for example, Henson, 2007).

However, if our results were detrimentally unreliable due to low event frequency, variance in scene length, limited scanning time or other factors typically enhanced to optimize the fMRI design, we would expect to have been unable to replicate our previous findings. The results presented here on the other hand show a near-identical pattern of activation as our previous results. Further, using multivariate pattern analysis techniques reported elsewhere we have been able to predict intrusive memories solely from the brain activity during encoding of film footage with traumatic content (Clark *et al.* 2014). Overall, this suggests that these fMRI results underlying intrusive memory encoding, while not ideal in all respects, are reliable in terms of replicability.

The prospective design and use of a scanner at encoding rely upon an analogue of trauma, and this is not the same as experiencing real trauma. However, repeated exposure to media film images of traumatic events have been associated with higher scores on the PTSD Checklist – Civilian Version (a measure of PTSD symptoms) (Silver *et al.* 2013) and higher acute stress symptoms (Holman *et al.* 2014). The inclusion of trauma exposure through electronic media, television and movies in the line of work in the new DSM-5 (American Psychiatric Association, 2013) also prompts the need for greater understanding of these forms of exposure.

Our intrusive memory involuntary recall task in the scanner has only been tested here at a time soon after the analogue trauma. Recent evidence suggests that immediate (1 h) and delayed (1 week) intrusive memories may result from different types of retrieval mechanisms (Staugaard & Berntsen, 2014). Immediate intrusive memories may relate more to salient aspects of the memorability at encoding (e.g. vividness, emotionality, recency), whereas delayed intrusive memories may reflect the influence of retrieval cues in the environment that elicit such involuntary recall. Our current results are on immediate intrusive memories. Future research should test a larger time interval by returning participants to the scanner at 1 week.

This study aimed to provide the first capture of the neural processes involved at the moment of intrusive memory involuntary recall. However, our intrusive memory involuntary recall analysis presents only the first steps in what will need to be a longer line of enquiry. Our control condition (button press alone) was used to subtract brain activity associated with the button press itself in the absence of an intrusion (for related methodology to capture neural activity associated with the occurrence of a hallucination in schizophrenia via balloon press, see Diederen *et al.* 2010; Hoffman *et al.* 2011). Future investigations should develop improved and appropriately powered control conditions to develop methods to capture intrusiveness, as this is key to many psychiatric phenomena. Additionally, further studies which specifically contrast voluntarily and involuntary recall, in particular following movie stimuli, are clearly required.

Finally, it is not possible to ascertain whether the intrusions are ‘truly’ spontaneous, or merely reported as spontaneous. Interestingly, this issue applies equally to the clinical form of this experimental analogue, since patients with PTSD are asked to report or monitor their spontaneously occurring intrusive memories during assessment/treatment. Future studies might seek to examine this issue further.

## Conclusions

Our analyses suggest that whilst experiencing trauma the brain behaves differently during moments that later become intrusive memories, consistent with clinical suggestions that the peritraumatic phase is important in predicting PTSD (Ozer *et al.* 2003; American Psychiatric Association, 2013). Whereas a strikingly widespread pattern of activation was involved at encoding, the left IFG was the only region involved in both the encoding and involuntary recall of intrusive memories. What are the clinical implications? Tentatively we suggest that if left IFG activation can be modulated during the encoding of trauma memory and its consolidation (Walker *et al.* 2003), then we may be able to modulate intrusive memory occurrence by reducing left IFG activation. Further, due to the association between the left IFG and language processing (Vigneau *et al.* 2006), results may provide a clue as to why certain talking-based interventions (e.g. critical incident stress debriefing) soon after trauma have been found to be detrimental (Roberts *et al.* 2009). Talking-based interventions increasing left IFG activity may serve to increase intrusive memory (re)encoding at this early time point. We note that at later time points (e.g. 1 month and later) trauma-focused cognitive–behavioural therapy is effective (e.g. Bisson *et al.* 2013).

In summary, after witnessing a traumatic event, it is only certain moments from the trauma that reappear as intrusive memories. Why it is that some moments rather than others become intrusive memories has long been a puzzle. We suggest that alterations in brain activation at the time of viewing trauma determine which moments will later become intrusive memories. In particular, activity in the left IFG seems to be key for both the encoding and the involuntary recall of intrusive memories. Further, we speculate that rather than disrupted encoding resulting in memory fragments and intrusive memories, a theoretical alternative is that intrusive memories result from better encoded memories at specific points in time.

## Supplementary material

For supplementary material accompanying this paper visit http://dx.doi.org/10.1017/S0033291715002007. click here to view supplementary material
