# Supplementary material for: Intrusive memories to traumatic footage: the neural basis of their encoding and involuntary recall
Source: Psychol Med. 2016 Feb;46(3):505–18. doi: 10.1017/S0033291715002007 (PMC4697303; doi:10.1017/S0033291715002007)
Supplement: Supplementary file 1 [file S0033291715002007sup001.doc]

**Supplementary material**

**Supplementary Method**

**Participants unable to be analysed**

Data could not be analysed for 6 participants. Two participants reported 0 intrusive memories, 1 had insufficient performance on the recognition memory test. For 3 further participants full data was not acquired due to 1 stopping the scan during film viewing, 1 failing to return to follow up and for 1 technical issues stopped the scan before film completion. These 6 participants did not differ on age [mean age = 21.0 years, *SD =* 2.53; t(39) = .46, *p* = .65], or gender [3 female, 3 male; χ^2^ = 3.23, *p* = .072] to the final sample.

**Baseline and mood measures**

Participants completed baseline measures (Beck Depression Inventory II[^1^](#_ENREF_1) and State-Trait Anxiety Inventory Trait version[^2^](#_ENREF_2)) followed by 10cm visual analogue scale mood ratings for ‘sad’, ‘hopeless’ and ‘depressed’.

**Recognition memory test**

The recognition memory test consisted of 201 stills, 103 stills were taken from the film (51 from control scenes) and 98 additional stills were selected to be foils. Foils were from unused sections of the film clips that had been edited out or from similar situations. Film stills were at least 6 seconds apart and were unique to all other points in the film. Stills were each presented for a maximum of 3 seconds and participants responded as to whether the recognised the still from the film they saw the previous week (yes/no decision). Upon response participants rated their confidence as *High Confidence, Low Confidence or Guessing*. Any correct answers rated as *Guessing* were excluded from the analysis.

**Additional participants recruited for control of intrusive memory involuntary recall**

Nine additional participants were recruited to act as controls for the intrusive memory involuntary recall analysis. The 9 participants did not differ from those that took part the main experiment on age [mean age = 24.22 years, *SD =* 3.46; t(42) = .69, *p* = .49] or gender [5 female, 4 male; χ^2^ = 3.04, *p* = .081].

**Supplementary Results**

**Baseline measures and mood change**

The mean BDI-II score was 4.26 (*SD* = 5.19) and the mean STAI-T score was 38.66 (*SD* = 9.63). A composite negative mood response was calculated from the mean of the three mood scales[^3^](#_ENREF_3); repeated measures ANOVA showed that negative mood significantly increased from pre (mean = 1.05, *SD =* 1.26) to post film viewing (mean = 3.14, *SD =* 1.94) [F(1,34) = 56.10, *p* < .001, η_p_^2^ = .62].

**Recognition memory test**

Signal detection theory was used to analyse the recognition memory test. Participants had a mean hit rate (correctly recognizing stills from the film) of .65 (*SD=* .11) and a mean false alarm rate (identifying foils as from the film) of .29 (*SD* = .11). Participants had a reasonable ability to discriminate between film stills and foils; mean d’ of .99 (SD = .27), but no bias towards choosing either; mean C of .079 (SD = .29).

For the stills analysis; the mean number of Intrusive recognized stills was 7.8 (*SD* = 3.73) and Potential recognized stills was 25.66 (*SD* = 7.34).

To test whether using stills (from the recognition memory test) instead of scenes (as in the first intrusive memory encoding analysis) did not change the pattern of results, we performed the same intrusive memory encoding analyses using only the stills. This revealed a similar pattern of results as to the scenes analysis, but with decreased spatial extent (Supplementary Figure 1).

**Supplementary Table S1.** “Potential” scenes length and position in the trauma film

| Clip | Scene Description | Duration (secs) |
| --- | --- | --- |
| 1 | Dismembered head | 5 |
| 1 | Leg sticking up out of car | 7 |
| 1 | Fire fighter carrying baby | 18 |
| 3 | Eye Operation | 32 |
| 4 | Man trapped in grey car | 16 |
| 4 | Woman trapped in van | 18 |
| 4 | Woman faints in van | 22 |
| 4 | Leg wound | 31 |
| 5 | Car hitting the boy | 23 |
| 6 | Body sticking out of car | 19 |
| 6 | Bodies moved and covered | 9 |
| 6 | Firemen lifting bodies | 16 |
| 8 | Leg operation | 33 |
| 9 | Bodies on the street | 26 |
| 9 | Bodies being placed into coffins | 33 |
| 10 | Shaving cut across throat | 30 |
| 11 | Student being intubated | 37 |
| 12 | Slow motion ricocheting of boy inside the car injuring the others | 27 |
| 14 | Elephant mauling keepers | 34 |
| 15 | Couple pinned to wall | 13 |

**Supplementary Table S2.** “Control” scenes length and position in the trauma film

| Clip | Scene Description | Duration (secs) |
| --- | --- | --- |
| 1 | Arrival of Fire truck | 11 |
| 2 | Eye make up 1 | 15 |
| 2 | Eye make up 2 | 13 |
| 2 | Eye make up 3 | 15 |
| 4 | St John’s ambulance men | 7 |
| 4 | Fire men with equipment to try and rescue woman from van | 15 |
| 4 | Paramedics checking the man’s non damaged leg | 16 |
| 5 | Boy playing in the garden | 26 |
| 7 | Nurses in operating theatre 1 | 26 |
| 7 | Nurses in operating theatre 2 | 36 |
| 7 | Nurses in operating theatre 3 | 13 |
| 10 | Man shaving as normal | 13 |
| 12 | Couple in town square before meeting friends | 14 |
| 13 | Elephants performing in circus tent | 32 |
| 15 | Couple on wall | 6 |
| 15 | Girl in hospital bed | 5 |

**Supplementary Fig. S1.** Whole brain analysis of Intrusive vs. Potential vs. Control stills as used in the recognition memory test. A similar pattern of activation can be seen but to a decreased spatial extent as for the whole brain intrusive memory encoding analysis.


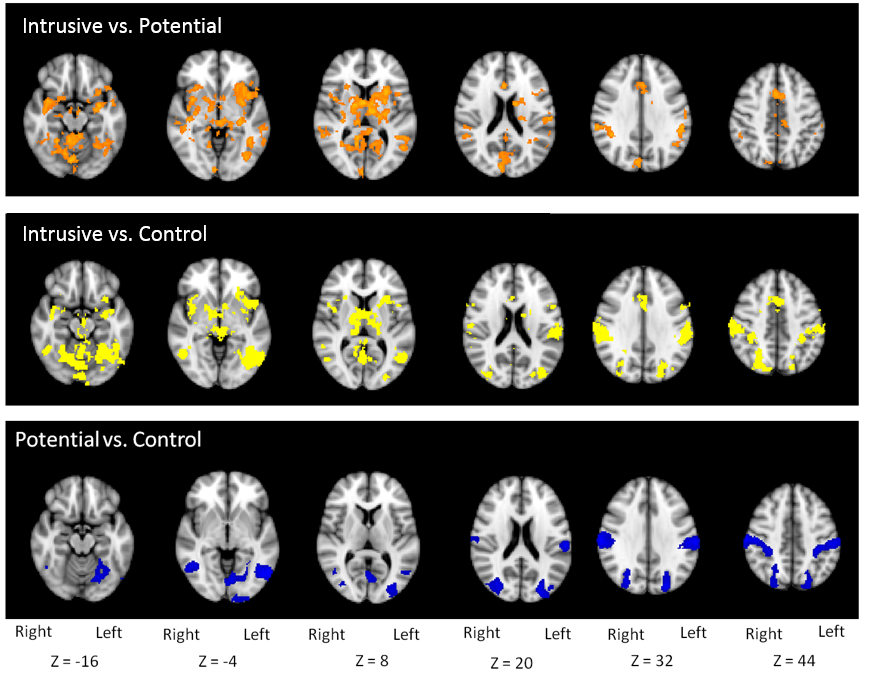


**References**

1. Beck AT, Steer RA, Brown GK. *Manual for the Beck Depression Inventory-II.* San Antonio, TX: Psychological Corporation; 1996.

2. Spielberger CD, Gorsuch RL, Lushene R, et al. *Manual for State-Trait Anxiety Inventory.* Palo Alto, CA: Consulting Psychologists Press; 1983.

3. Holmes EA, James EL, Kilford EJ, et al. Key steps in developing a cognitive vaccine against traumatic flashbacks: visuospatial Tetris versus verbal Pub Quiz. *PLoS ONE.* 2010;5(11):e13706.
